# Supplementary material for: Validating an Assessment Tool for Oral Health and Oral Care Procedures Performed by Healthcare Workers for Older Residents in Long-Term Care Institutions
Source: Healthcare (Basel). 2024 Feb 28;12(5):558. doi: 10.3390/healthcare12050558 (PMC10930508; doi:10.3390/healthcare12050558)
Supplement: Supplementary file 1 [file healthcare-12-00558-s001.zip › healthcare-2843682-supplementary.pdf]

Table S1. Expert review results for relevancy check and comments

Question No.	Item Questions	Interpretation	Recommendation
OHAQ1	Lips: redness/whiteness/pain/dryness/crusting/chapped/enlargement/irregularities/ulcer/blister/bleeding/Not applicable	Relevant	• Change "whiteness" to "pale"<br>• Delete "not applicable"
OHAQ2	Tongue: redness/pale/discoloration/pain/dryness/smoothness/enlargement/irregularities/ulcer/blister/bleeding/Not applicable	Relevant	• Change "whiteness" to "pale"<br>• Delete "not applicable"
OHAQ3	Oral mucosa: redness/pale/pain/dryness/bleeding/ulcer/outgrowth/not applicable	Relevant	• Change "whiteness" to "pale"<br>• Delete "not applicable"
OHAQ4	Gum: redness/pale/pain/dryness/bleeding/ulcer/enlargement/ not applicable	Relevant	• Change "whiteness" to "pale"<br>• Delete "not applicable"
OHAQ5	Tooth: count (<20/<10/nil)/ tartar/deposits/plaque/looseness/discomfort/pain/cavity/caries/severe wear/failing restoration/ not applicable	Relevant	• Added ">20"<br>• Delete "not applicable"
OHAQ6	Denture(s): tartar/deposits/plaque/severe wear/looseness/discomfort/pain/poor color/ not applicable	Relevant	• Delete "not applicable"
OHAQ7	Oral hygiene: bad breath/food debris between teeth	Relevant	Add this item for oral hygiene
OCPQ1	Provide explanations to older resident about care during oral care.	Relevant	Rephrased as "Explain the oral care procedure to the older resident during the procedure."
OCPQ2	Assist older resident to appropriate position, such as sitting upright with adequate neck support or lying aside for the oral care procedure.	Relevant	Rephrased as "Position the older resident properly during oral care procedures such as sitting upright with adequate neck support or lying aside if no contraindications."
OCPQ3	Ensure visibility of oral condition for accessibility for oral care and oral assessment.	Relevant	Rephrased as "Ensure the oral cavity is visible under appropriate lighting for oral care and assessment."
OCPQ4	Perform initial oral health assessment.	Relevant	Add (Please complete the oral health assessment section)
OCPQ5	Place protective sheet under the chin or the face for the resident.	Relevant	Moved after OCPQ3
OCPQ6	Have oral care equipment ready, such as toothbrush with toothpaste, dental floss, spongy sticks for oral care.	Relevant	Rephrased as "Prepare the equipment for oral care aids such as toothbrushes, toothpaste, interdental cleaning aids, and spongy sticks for oral care and cleaning."
OCPQ7	Perform oral care thoroughly.	Relevant	Rephrased as "Perform oral care thoroughly, including tongue, teeth, gum, oral membrane, and lips, and apply lip moisturizer if appropriate."

OCPQ8	Observe abnormalities during care such as pain and bleeding.	Relevant	Rephrased as "Observe any abnormality during the oral care procedure such as pain and bleeding."
OCPQ9	Ensure safety during oral care.	Relevant	Rephrased as "Ensure the safety of the resident and the healthcare provider during oral care."
OCPQ10	Use standard precautions and appropriate infection control measures during oral care.	Relevant	Moved to OCPQ2
OCPQ11	Tidy up after care	Relevant	Rephrased as "Tidy up after oral care."
OCPQ12	Remove dentures if present.	Relevant	Added this item to increase applicability
OCPQ13	Report any oral abnormalities immediately to the in charge	Relevant	Added this item to increase applicability
OCPQ14	Document all the assessments and care	Relevant	Added this item to increase applicability

NOTE: Number of items considered relevant and included by all experts, n = 6.

Table S2. The CVI and CVR results

Question No.	Item Questions	CVR	Recommendations	I-CVI	Interpretation	Recommendations
OHAQ1	Lips: redness/pale/pain/dryness/ crusting/chapped/enlarge-ment/irregularities/ulcer/ blister/bleeding	1.00	Included	1.00	Relevant	Included
OHAQ2	Tongue: redness/pale/discoloration/ pain/dryness/smoothness/ enlargement/irregularities/ ulcer/blister/bleeding	1.00	Included	1.00	Relevant	Included
OHAQ3	Oral mucosa: redness/pale/pain/dryness/ bleeding/ulcer/outgrowth	1.00	Included	1.00	Relevant	Included
OHAQ4	Gum: redness/pale/pain/dryness/ bleeding/ulcer/enlargement	1.00	Included	1.00	Relevant	Included
OHAQ5	Tooth: count (>20/<20/<10/nil)/ tartar/deposits/plaque/ looseness/discomfort/pain/ cavity/caries/severe wear/failing restoration	1.00	Included	1.00	Relevant	Included
OHAQ6	Denture(s): tartar/deposits/plaque/ severe wear/looseness/ discomfort/pain/poor color/ not applicable	1.00	Included	1.00	Relevant	Included
OHAQ7	Oral hygiene: bad breath/food debris between teeth	1.00	Included	1.00	Relevant	Added
OCPQ1	Explain the oral care procedure to the older resident during the procedure.	1.00	Included	1.00	Relevant	Included
OCPQ2	Use standard precaution and appropriate infection control measures during oral care procedures.	1.00	Included	1.00	Relevant	Included
OCPQ3	Position the older resident properly during oral care procedures such as sitting upright with adequate neck support or lying aside if no contraindications.	1.00	Included	1.00	Relevant	Included
OCPQ4	Place a protective sheet under the older resident's chin or the face.	1.00	Included	1.00	Relevant	Included
OCPQ5	Ensure the oral cavity is visible under appropriate lighting for oral care and assessment.	1.00	Included	1.00	Relevant	Included
OCPQ6	Perform initial oral health assessment. (Please complete the oral health assessment section)	1.00	Included	1.00	Relevant	Included
OCPQ7	Prepare the equipment for oral care aids such as toothbrushes, toothpaste, interdental cleaning aids,	1.00	Included	1.00	Relevant	Included

	and spongy sticks for oral care and cleaning.					
OCPQ8	Remove dentures if present.	1.00	Included	1.00	Relevant	Added
OCPQ9	Perform oral care thoroughly, including tongue, teeth, gum, oral membrane, and lips, and apply lip moisturizer if appropriate.	1.00	Included	1.00	Relevant	Included
OCPQ10	Observe any abnormality during the oral care procedure such as pain and bleeding.	1.00	Included	1.00	Relevant	Included
OCPQ11	Ensure the safety of the resident and the healthcare provider during oral care.	1.00	Included	1.00	Relevant	Included
OCPQ12	Tidy up after oral care.	1.00	Included	1.00	Relevant	Included
OCPQ13	Report any oral abnormalities immediately to the in charge.	1.00	Included	1.00	Relevant	Added
OCPQ14	Document all the assessments and care.	1.00	Included	1.00	Relevant	Added

NOTE: Number of experts evaluated the item essential. CVR = (Ne − N/2)/(N/2) with 6 experts (n = 6), items with the CVR bigger than 0.99 remained in the questionnaire.

Table S3 Assessment tool for oral health and oral care procedures

Part I. Oral Health Assessment

Assessment Items	Please circle the appropriate box (you can circle more than one item)						Describe your circle item(s) if any
1. Lips	Normal (Pink)	Redness/ pale	Pain	Dryness/ crusting/ chapped	Enlargement/ irregularities	Ulcer/ blister/ bleeding	
2. Tongue	Normal (Pink)	Redness/ pale/ discoloration	Pain	Dryness/ smoothness	Enlargement/ irregularities	Ulcer/ bleeding	
3. Oral mucosa	Normal (Pink)	Redness/ pale	Pain	Dryness	Bleeding	Ulcer/ outgrowth	
4. Gum	Normal (Pink)	Redness/ pale	Pain	Dryness	Bleeding	Ulcer/ enlargement	
5. Teeth	Normal & Count >20	Count: <20/ <10/ nil	Tartar/ deposits	Plaque	Looseness	Discomfort/ Pain	
		Cavity/ caries	Severe wear	Failing restoration			
6. Denture(s)	Normal[¥]	Tartar/ deposits	Plaque	Severe wear	Looseness	Discomfort/ Pain	
		Poor color					
7. Oral hygiene	Good	Bad breath	Food debris between teeth				

[¥] Older residents reported daily use of dentures to eat and speak; and denture(s) fits reasonably well in the mouth and does(do) not appear to be faulty/defective.

Need to see a dentist: No Yes

Dentist to be referred: ______________________ Appointment booked on: ____________

Staff name & signature: ______________________ Date: ____________

Part II. Assessment of Oral Care Practice of Healthcare Workers to An Older Resident

No.	Items	Unsatisfactory	Satisfactory	Not done	Remarks
1	Explain the oral care procedure to the older resident during the procedure.				
2	Use standard precaution and appropriate infection control measures during oral care procedure.				
3	Position the older resident properly during oral care procedure such as sitting upright with adequate neck support or lying aside if no contraindications.				
4	Maintain communication with the older resident during the procedure.				
5	Place a protective sheet under the older resident's chin or the face.				
6	Ensure the oral cavity is visible under appropriate lighting for oral care and assessment.				
7	Perform initial oral health assessment.<br>(Please complete the oral health assessment form)				
8	Prepare the equipment for oral care aids such as toothbrushes, toothpaste, interdental cleaning aids, and spongy sticks for oral care and cleaning.				
9	Remove denture(s) if present and provide appropriate denture hygiene and care.<br>(Removed denture(s) during oral hygiene assessment shall be cleaned by a soft bristle toothbrush with mild soap solution, thoroughly rinsed before reinserted into mouth afterwards.)				
10	Perform oral care thoroughly, including cleaning the soft tissues (tongue, gum, oral membrane, and lips), and brushing/ flossing the teeth, wiping the soft tissue with a moist gauze, and apply lip moisturizer if appropriate.				

11	Observe any abnormality during the oral care procedure such as pain and bleeding.				
12	Ensure the safety of the resident and the healthcare provider during oral care.				
13	Tidy up after oral care.				
14	Provide brief education and/or oral condition to the older resident after the procedure.				
15	Report any oral abnormalities immediately to the in charge.				
16	Document all the assessments and care.				

Overall comments: ______________________________

____

____

Name of responsible staff: ____________________ Signature of responsible staff: ____________________

Name of assessor (if applicable): ______________ Signature of assessor (if applicable): ______________

Date and Time: ______________________________
